# Leadership Styles Among Nurses: A Cross-Sectional Analysis Within the Full Range Leadership Framework

**DOI:** 10.3390/nursrep16040141

**Published:** 2026-04-15

**Authors:** Cátia Moreira, Pedro Moutinho, Paulo Alves, Liliana Mota

**Affiliations:** 1 Center for Interdisciplinary Research in Health, Faculty of Health Sciences and Nursing, Universidade Católica Portuguesa, 4169-005 Porto, Portugal; s-ctmoreira@ucp.pt (C.M.); pjalves@ucp.pt (P.A.); 2 Independent Researcher, 4435-492 Porto, Portugal; pedrofilipeam@gmail.com; 3 Departamento de Enfermagem, Escola Superior de Saúde Norte da Cruz Vermelha Portuguesa, 3720-126 Oliveira de Azeméis, Portugal; 4 RISE-Health, Faculdade de Medicina da Universidade do Porto, 4200-319 Porto, Portugal

**Keywords:** leadership, nursing, leadership styles, full range leadership model, transformational leadership

## Abstract

**Background/Objective**: Leadership in nursing has been conceptualized as a multidimensional construct influencing organisational functioning and professional practice. Within the Full Range Leadership Model, leadership comprises transformational, transactional, and passive–avoidant dimensions that may coexist within individuals. This study aimed to examine how leadership dimensions coexist and interact among nurses and to explore their associations with professional characteristics within the FRLM framework. **Methods:** A cross-sectional quantitative study was conducted between November and December 2024 among 141 Portuguese nurses affiliated with a professional association dedicated to nursing leadership. Leadership behaviours were assessed using the Multifactor Leadership Questionnaire. A non-probability convenience sampling strategy was used. Descriptive and inferential analyses were performed using SPSS. **Results:** Transformational leadership emerged as the predominant behavioural pattern (M = 3.17, SD = 0.38), followed by transactional leadership (M = 2.51, SD = 0.46), with minimal laissez-faire behaviours (M = 0.83, SD = 0.50). Managers demonstrated significantly higher transformational scores (mean difference = 0.16, *p* = 0.018) and lower laissez-faire scores (mean difference = −0.27, *p* = 0.01) than specialists. Transformational leadership was positively correlated with transactional leadership (r = 0.309, *p* < 0.01) and negatively correlated with laissez-faire behaviours (r = −0.339, *p* < 0.01). **Conclusions:** The findings indicate a predominant transformational leadership profile among nurses, accompanied by complementary transactional behaviours and low passive–avoidant tendencies. The observed correlations support a dimensional interpretation of leadership consistent with the Full Range Leadership Model. These findings provide descriptive insight into leadership patterns within this nursing sample and may inform leadership development initiatives in comparable healthcare contexts.

## 1. Introduction

Leadership is widely recognised as a fundamental component of nursing practice and healthcare management. Nurse leaders play a crucial role in shaping professional practice, influencing organisational culture and promoting high standards of care delivery. The leadership style of nurses has been shown to directly influence team performance, patient and professional safety, quality, and health gains [1,2]. Effective leadership strategies have been shown to improve patient outcomes, increase job satisfaction among nursing professionals and boost the overall efficiency of healthcare organizations. Modern healthcare systems are characterized by increasing complexity, limited resources, and growing demands for quality and safety. In this context, the leadership style adopted by nurses assumes relevance, as it affects team cohesion and decision-making processes.

In healthcare, leadership is conceptualized as a dynamic social process through which an individual strategically influences others to achieve shared goals and enhance organisational outcomes. Recent research suggests that leadership involves the ability to direct, motivate, and support team members in complex and ever-changing environments [3]. In the field of health services, effective leadership is paramount to achieving optimal standards. This has implications for both healthcare professionals and patients [4]. Leadership is a topic that has been extensively researched because it is an essential skill for cultivating conducive work environments and empowering nurses to exert influence over their teams in pursuit of enhanced outcomes [5].

It is vital that leaders recognize that leadership style is a key element in the progress of increasing healthcare organisational productivity. Leadership styles refer to behavioural patterns that influence motivation, communication, and decision-making among followers. These leadership styles are shaped by the characteristics of leaders and contextual factors. In health and nursing research, relational leadership styles demonstrate distinct behavioural patterns that are associated with positive workforce outcomes, such as job satisfaction and work environment quality [1]. Leadership styles in nursing are influenced by a variety of factors, including individual leader characteristics such as prior experience, education, personal attributes, and participation in leadership development activities, as well as organisational and contextual factors such as the work environment, job expectations, and relational dynamics [1,5,6,7,8]. Collectively, these factors influence how leaders communicate, motivate, and engage their teams [9]. Healthcare organizations require a variety of leadership styles to function effectively and enhance healthcare outcomes. It is essential that leaders within these organizations possess the ability to think innovatively and demonstrate a high level of energy [10]. Over the past decade, there has been a significant increase in the volume of research conducted in the field of nursing leadership. A leader may exhibit a variety of leadership styles, characterized by behavioural dimensions. The focus of task-oriented leadership is on the coordination and assignment of work to followers [3]. The Full Range of Leadership Model (FRLM) is a model of leadership styles. It was developed by Avolio and Bass and provides a broad perspective [11,12].

The Full Range Leadership Model conceptualises leadership as comprising three broad dimensions: transformational, transactional, and passive–avoidant leadership [11,12]. Transformational leadership refers to behaviours that inspire, intellectually stimulate, and individually support followers, thereby promoting engagement and change. Transactional leadership refers to more exchange-based behaviours, such as clarifying expectations, monitoring performance, and reinforcing goal attainment through contingent reward. Passive–avoidant leadership reflects the relative absence or delay of leadership action and includes behaviours such as passive management by exception and laissez-faire leadership. Within the FRLM, these dimensions are not understood as mutually exclusive categories; rather, they may coexist to different degrees within the same professional context or individual leader profile.

Recent nursing research supports the use of the Full Range Leadership Model as an analytical framework rather than a purely descriptive one. In particular, transformational leadership—and especially its components of idealised influence, inspirational motivation, intellectual stimulation, and individualised consideration—has been associated with higher nurse work engagement, stronger motivation, greater job satisfaction, higher organisational and professional commitment, and more positive work environments [13,14,15]. Evidence also suggests that transformational leadership may contribute, both directly and indirectly, to healthier nursing work environments through mechanisms such as structural empowerment, organisational commitment, and job satisfaction [13]. Transactional leadership has shown more circumscribed but still meaningful associations, particularly with work engagement, role clarity, performance-based motivation, and adherence to structured care processes and standardised protocols [16,17]. By contrast, passive–avoidant leadership, including laissez-faire tendencies, has been associated with lower engagement, dissatisfaction, burnout, and greater turnover-related risk [14,17]. Taken together, these findings reinforce the relevance of examining FRLM dimensions as analytically distinct, yet potentially coexisting, leadership patterns in nursing research and practice.

Leadership in nursing is a key component of healthcare quality, directly impacting clinical outcomes, patient safety, professional satisfaction, and organisational efficiency. The current healthcare context is characterized by highly complex care, a shortage of human resources, and growing demands for accountability. The nurse leader plays a strategic role in team management and in promoting favourable professional practice environments [4]. The impact of leadership style on expected outcomes is significant. Analysing different leadership styles in nursing and their implications for healthcare is essential for understanding organisational phenomena and developing sustainable clinical governance models. Although nursing leadership has been extensively studied internationally, there is limited evidence on how leadership styles are enacted within specific healthcare contexts. This discrepancy hinders the formulation of effective, context-specific strategies for leadership, workforce management and enhancing patient care. Therefore, developing a nuanced understanding of leadership styles in nursing teams is essential to inform education, management and policy initiatives across diverse settings.

Although nursing leadership has been widely studied internationally [1,18], empirical research examining leadership styles within Portuguese nursing contexts remains comparatively limited [4]. Existing Portuguese evidence has highlighted the relevance of nurse leadership roles, but there is still limited contextual evidence on how leadership dimensions are distributed and combined among Portuguese nurses in leadership-related roles [4]. In this sense, the present study does not seek to establish a novel leadership pattern, but rather to provide contextual descriptive evidence from a Portuguese sample with leadership-related engagement.

In light of these considerations, the present study addresses the following research question: How are transformational, transactional, and passive–avoidant leadership styles distributed among nurses, and how are these dimensions interrelated within the Full Range Leadership Model framework? Accordingly, the objective of this study was to examine how leadership dimensions coexist and interact among nurses and to explore their associations with professional characteristics within the Full Range Leadership Model framework. 

## 2. Materials and Methods

### 2.1. Study Design

This study employed a descriptive cross-sectional design with correlational and group-comparison analyses to examine leadership styles among nurses in management contexts. For the purposes of this study, the STROBE reporting guideline [19] was used to draft this manuscript, and the STROBE reporting checklist [20] was used when editing.

### 2.2. Population and Sample

The study population comprised nurses from a Portuguese nursing association. This association was selected for its relevance at a national nursing level in the field of management and leadership. This selection ensures that the organisational context was appropriate for the quantitative approach adopted in the study of leadership styles. The association where the study took place was established in May 2009 and had 867 active members at the end of 2024, 70% of whom were women, with an average age of 53, the most common age group being between 46 and 65 [21].

The sampling technique was non-probability. All participants provided informed consent by voluntarily completing the data collection. The association’s directors invited the members of the association to participate in the study via email and WhatsApp. The information sheet outlined the objectives and scope of the study, emphasizing that participation was voluntary and that participants had the right to withdraw at any time and to remain anonymous. The survey link was distributed to the participants and was conducted online from November to December 2024.

Due to the descriptive nature of the study, all nurses from the association working in Portugal were eligible for inclusion in the study.

Data were collected using a structured self-administered questionnaire designed to assess leadership styles among nurses. The questionnaire was administered online, and all responses were anonymous and checked for completeness prior to data entry.

The study population comprised 867 nurses affiliated with the Portuguese Nursing Association who are involved in nursing management and leadership roles. All nurses who provided consent were included, resulting in a final sample of 141 participants. The final sample included nurses with a range of professional experiences and leadership-related roles; however, because recruitment was conducted through a leadership-focused professional association and a convenience sampling strategy was used, the sample should be interpreted as reflecting a specific leadership-engaged subgroup rather than the broader nursing population. The use of a convenience sampling strategy may limit the generalisability of the findings.

Of the 867 eligible members invited to participate, 198 returned the questionnaire, corresponding to a response rate of 22.8%. After data screening, 141 questionnaires were considered complete and valid for final analysis, representing 71.2% of returned questionnaires and 16.3% of the invited population [21].

### 2.3. Sample Size

Given the exploratory and descriptive nature of the study, and the use of an accessible professional population, the final sample was treated as an available convenience sample. A pragmatic sample size estimate based on Slovin’s formula was considered during study planning; however, this approach should be interpreted cautiously, as simplified formula-based estimates do not replace formal power analyses based on effect size assumptions and predefined primary outcomes [22]. Accordingly, the present study should be understood as descriptive and hypothesis-generating.

### 2.4. Instruments

The data collection instrument is composed of two sections. The first section concerns the subject’s socio-demographic characteristics, while the second part is the Multifactor Leadership Questionnaire (MLQ). The following variables were used to characterise the socio-demographic profile of the sample: gender, age, level of education, working experience, leadership experience, number of nurses, context of work and professional category. Leadership dimensions were assessed using the Portuguese version of the Multifactor Leadership Questionnaire (MLQ-5X), administered as a self-report online questionnaire [11].

The MLQ-5X comprises 45 items divided into nine subscales to capture a broad range of leadership behaviours. The MLQ-5X is grounded in the Full Range Leadership Model (FRLM) and assesses leadership behaviours across three core dimensions: transformational (relation and change focused), transactional (task-focused) and passive–avoidant (absence of leadership) [11]. Items are scored using a five-point Likert scale, with responses ranging from “never” (0) to “frequently, if not always” (4) [11]. These ratings reflect the degree to which certain leadership behaviours are present and are based on the components of the FRLM of Avolio and Bass [11]. The model incorporates the following leadership styles, ranked from most to least effective: transformational, transactional and passive–avoidant [11]. The MLQ-5X manual provides a scoring key that indicates which scale items should be grouped to interpret the acquired data. There are twenty items used to measure transformational leadership, eight items used to measure transactional leadership and eight items used to measure passive–avoidant leadership. To measure leadership outcomes, three items are used to assess extra effort. Effectiveness is measured using four items, while satisfaction is measured using two items. These are considered results of leadership behaviour [11]. More detailed information about the questionnaire is provided in the MLQ-5X Manual. This is due to copyright regulations. The same applies to the scoring key and to information about which items are related to which leadership style [11]. The FRLM is a well-established model, supported by more than 35 years of accumulated evidence on its validity [12].

Cronbach’s alpha was used to assess the internal consistency of the MLQ-5X, with a value of ≥0.80 considered good and <0.60 considered poor [23]. Research has demonstrated that the MLQ-5X possesses adequate construct and predictive validity, in addition to meeting the model fit requirement. The original scale exhibited internal consistency greater than 0.78 for all items, with the exception of the active management by exception item, which registered at 0.64 [11]. To maintain reliability, standardised data collection procedures were implemented. All responses were checked for completeness and accuracy prior to coding.

The present article focused on the 36 MLQ-5X items corresponding to transformational, transactional, and passive–avoidant leadership dimensions. The leadership outcome items included in the full 45-item instrument were not analysed in this study.

In the present study, the analyses focused on the 36 MLQ-5X items corresponding to the leadership dimensions examined. For these 36 items, internal consistency was acceptable (Cronbach’s alpha = 0.774). For reference, the full 45-item version of the instrument showed good overall internal consistency in the present sample (Cronbach’s alpha = 0.845). Internal consistency estimates for the main leadership dimensions were as follows: transformational leadership (α = 0.846), transactional leadership (α = 0.569), and passive–avoidant leadership (α = 0.801). At the subscale level, Cronbach’s alpha values were 0.352 for idealised influence attributes, 0.493 for idealised influence behaviours, 0.805 for inspirational motivation, 0.609 for intellectual stimulation, 0.648 for individual consideration, 0.504 for contingent reward, 0.629 for management-by-exception active, 0.559 for management-by-exception passive, and 0.745 for laissez-faire. These results indicate stronger internal consistency for the broader transformational and passive–avoidant dimensions than for several individual subscales, which should be considered when interpreting subscale-level findings.

Ethical approval was obtained from the relevant institutional review board, and informed consent was obtained from all participants. Measures were implemented to ensure the anonymity and confidentiality of study participants.

### 2.5. Analysis

The statistical analysis employed both descriptive and inferential statistics using the Statistical Package for the Social Sciences (SPSS) program version 31.0.1.0.

Questionnaires were screened for completeness, coded, and entered into the SPSS for analysis. All data were coded and securely stored on the server, accessible only to the researcher via a password-protected account for subsequent statistical analysis. Responses with missing values in the characterisation variables were retained and treated using the pairwise deletion method in the relevant analyses. Responses with missing values in the scales were eliminated (listwise deletion), as this prevented the construction of scores. Accordingly, 57 returned questionnaires were excluded from the final analytic sample because missing values in the scale items prevented score construction, resulting in 141 valid cases for analysis.

The data were summarised using descriptive statistics, including frequencies, percentages, means and standard deviations. Frequencies and percentages were used to describe the categorical data, while means with standard deviations (SD) were used for the continuous data. Leadership dimensions were assessed using the MLQ. Scores were interpreted using a descriptive tendency approach, reflecting relative predominance rather than fixed leadership categories. Leadership dimensions were primarily analysed as continuous and non-mutually exclusive constructs, consistent with the FRLM [11,12]. As a secondary descriptive procedure, an additional classification based on the highest mean dimension score was used only to summarise relative predominance among participants and should not be interpreted as indicating fixed or exclusive leadership categories. Prior to inferential analyses, the assumptions of normality and homogeneity of variance were assessed. Inferential analyses were performed to examine the relationships between leadership dimensions, as assessed using the MLQ (pure and tendency-based scores), and participant characteristics. An evaluation was conducted to ascertain the differences in leadership style scores according to gender, professional category and academic qualification. To this end, independent-samples *t*-tests were used to analyse the data, while Pearson’s correlation coefficient was utilised to assess the correlation with age. The assumption of normality was assessed using the Kolmogorov–Smirnov test (with Lilliefors correction) and the absolute values of skewness and kurtosis [12]. Levene’s test was used to evaluate the homogeneity of variances across different groups. To mitigate the occurrence of false positives due to multiple testing, only the aforementioned variables were examined as potential predictors of leadership dimensions. Nevertheless, given the exploratory nature of subscale-level analyses, results should be interpreted with caution due to the increased risk of Type I error. Additionally, the relationships between leadership dimension scores were explored using Pearson correlations and visualised via a scatterplot matrix across leadership style scales. For all inferential analyses, statistical significance was set at 0.05. Given the exploratory and descriptive aims of the study, and considering the final sample size, the analytical strategy was intentionally conservative. More advanced analytical approaches, such as multivariable regression, cluster analysis, or profile-based modelling, may provide additional insight into the coexistence of leadership dimensions, but these were considered beyond the scope of the present study. Because multiple subgroup and subscale comparisons were performed, the possibility of Type I error must be considered. For this reason, these analyses should be interpreted as exploratory, and emphasis should be placed on the consistency and magnitude of the observed patterns rather than on isolated *p*-values [23].

### 2.6. Ethical Considerations

All participants were informed about the purpose and procedures of this survey on the first page of the questionnaire. There was no power relationship between the investigators and the participants, and no pressure or inducement was applied. The participants were informed that their involvement in the study was entirely voluntary and anonymous, and that they could withdraw at any stage without facing any negative repercussions. They were also assured that all data would be kept confidential through secure storage at the Catholic University, accessible only to researchers involved in the project. Following the signing of the electronic informed consent form, the participants proceeded to complete the questionnaire on mobile devices or computers.

The Ethics Committee of the Catholic University approved this study (number 44/2024) and the rights to use the instrument were duly acquired from the publisher. The study was also conducted in accordance with the Helsinki Declaration.

## 3. Results

### 3.1. Sample Characteristics

Of the 198 returned questionnaires, 141 were complete and valid for final analysis. This corresponded to 22.8% of the 867 invited members and 71.2% of all returned questionnaires. Of the sample of nurses, 110 were female (78.0%) and 31 were male (22.0%). The characteristics of the participants are provided in Table 1. The average age in the sample was 52.50 years (SD = 7.92), ranging from 30 to 66 years. Of the participants, 10 had a PhD (7.1%), 78 had a master’s degree (55.3%), and 53 had a bachelor’s degree (37.6%). Of the participating nurses, 136 held the title of specialist from the Order of Nurses (96.5%). Forty nurses (28.4%) specialised in medical-surgical nursing, 34 in rehabilitation (24.1%), 24 in public health, community and family health nursing (17.0%), 18 in child and paediatric health nursing (12.8%), 10 in mental health and psychiatric nursing (7.1%), with the lowest frequency in the area of maternal and obstetric health (9, 6.4%). Of the participants, 127 had postgraduate training in management, leadership and administration (90.1%), but only 89 (63.1%) had additional management skills certified by the Portuguese Nursing Order.

Among the participants who held management positions, the average experience in this role was found to be 12.74 years (SD = 8.85), with a range from 0 to 32 years. Regarding the number of nurses in the team they led, participants reported an average of 34.33 (SD = 51.88), ranging from 3 to 500 nurses. Of the nurses who participated, 85 worked at the operational management level (60.3%), 15 at the tactical level (10.6%), and 16 at the strategic level (11.3%).

In terms of employer sector, 134 individuals were employed in the public sector (95%), 4 in the private sector (2.8%), and 3 in the public–private sector (2.1%). The hospital sector was the most common area of work, with 97 respondents (68.8%), followed by primary health care with 29 (20.6%), other health areas with 9 (6.4%) and teaching with 6 (4.3%).

### 3.2. Distribution of Leadership Styles

Descriptive statistics for leadership dimensions are presented in Table 2.

Transformational leadership showed the highest mean score among the three broad leadership dimensions (M = 3.17, SD = 0.38), followed by transactional leadership (M = 2.51, SD = 0.46), whereas passive–avoidant leadership presented the lowest mean values (M = 0.83, SD = 0.50). This pattern is interpreted as indicating relative predominance at the group level rather than mutually exclusive leadership categories.

At the subdimension level, individual consideration showed the highest mean score among transformational components (M = 3.40, SD = 0.48), followed by behaviours of idealised influence (M = 3.25, SD = 0.47), inspirational motivation (M = 3.23, SD = 0.56), and intellectual stimulation (M = 3.14, SD = 0.46). Attributes of idealised influence presented comparatively lower values (M = 2.83, SD = 0.50). Within the transactional dimension, contingent reward showed higher mean values (M = 2.86, SD = 0.57) compared to active management by exception (M = 2.16, SD = 0.64). Passive management by exception (M = 1.01, SD = 0.53) exceeded laissez-faire behaviours (M = 0.65, SD = 0.55) within the passive–avoidant dimension.

To improve clarity, leadership dimensions and subdimensions are presented in accordance with the hierarchical structure of the Full Range Leadership Model. In particular, passive–avoidant leadership is presented as a broader dimension that includes passive management by exception and laissez-faire behaviours.

Given that leadership dimensions are conceptualised as non-mutually exclusive behavioural patterns, an additional classification approach was conducted based on each participant’s highest mean dimension score. Using this criterion, 132 participants (93.6%) presented higher transformational scores, whereas 9 participants (6.4%) presented higher transactional scores. No participant demonstrated passive–avoidant leadership as the highest scoring dimension.

### 3.3. Differences in Leadership Dimensions by Gender

Independent-samples *t*-tests were conducted to examine differences in leadership dimensions according to gender (Table 3). No statistically significant differences were observed between female and male participants across transformational, transactional, or passive–avoidant leadership dimensions (all *p* > 0.05). Mean differences were small across all subdimensions.

### 3.4. Associations Between Age and Leadership Dimensions

Pearson correlation analyses indicated no statistically significant associations between age and the broader leadership dimensions. Small negative correlations were observed for attributes of idealised influence (r = −0.174, *p* = 0.039) and passive management by exception (r = −0.231, *p* = 0.006); however, given the low to modest internal consistency of these subscales, these findings should be interpreted cautiously and considered exploratory. No other age-related associations reached statistical significance (*p* > 0.05), Table 4.

### 3.5. Differences in Leadership Dimensions by Professional Category

Nurse managers showed higher mean scores in transformational leadership (*p* = 0.018) and intellectual stimulation (*p* = 0.011), whereas specialist nurses showed higher mean scores in passive–avoidant leadership (*p* = 0.010), passive management by exception (*p* = 0.005), and laissez-faire behaviours (*p* = 0.039) (Table 5). No statistically significant differences were observed for transactional leadership or the remaining subdimensions (*p* > 0.05). Although statistically significant, these differences were of small-to-moderate magnitude and should be interpreted cautiously, particularly at the subscale level, given the exploratory nature of the analyses and the lower internal consistency observed for some subscales.

### 3.6. Differences in Leadership Dimensions by Academic Qualification

Differences according to academic qualification are presented in Table 6. Participants with doctoral degrees showed higher mean scores for behaviours of idealised influence compared with participants with other academic qualifications (*p* = 0.014); however, this finding should be interpreted with particular caution because of the small number of participants with doctoral degrees and the low internal consistency of this subscale. No other leadership dimensions demonstrated statistically significant differences by academic qualification (*p* > 0.05; Table 6).

The difference observed in behaviours of idealised influence should be interpreted cautiously because of the small number of participants with doctoral degrees and the low internal consistency of this subscale. More broadly, these subgroup findings should be considered exploratory in light of the convenience sampling strategy and the possibility of Type I error due to multiple comparisons.

### 3.7. Associations Among Leadership Dimensions

**Figure 1:**
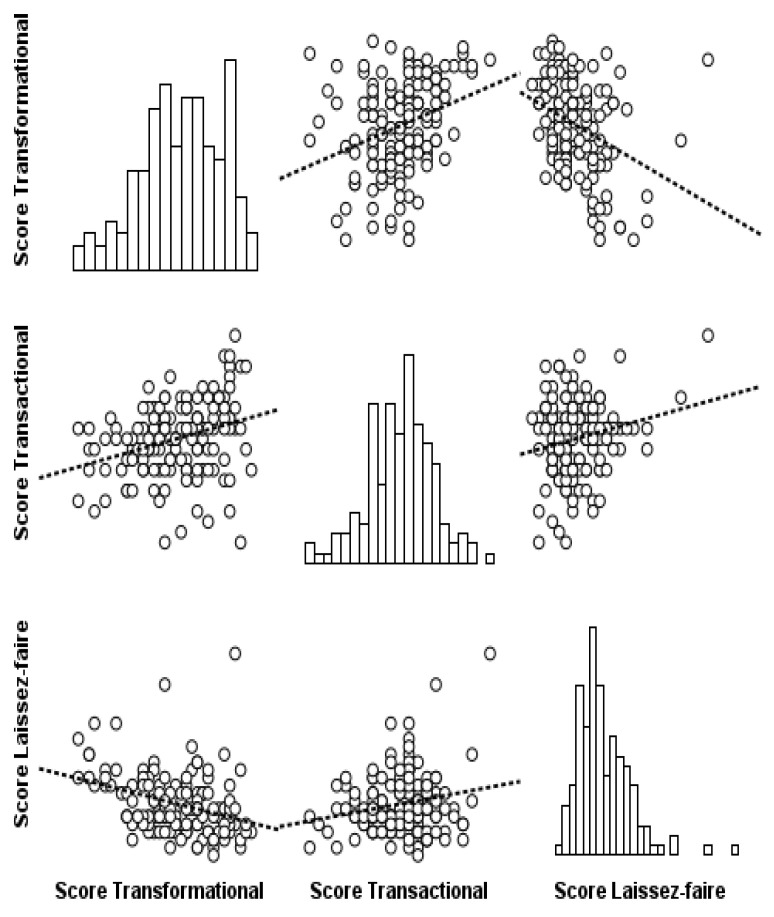
Matrix of scatter diagrams between leadership style scales. The best-fit equation, as determined by OLS regression, is illustrated by the dashed lines.

Associations among the primary leadership dimensions are illustrated in the scatterplot matrix (Figure 1). A moderate positive correlation was identified (r = 0.309, *p* < 0.01) between transformational and transactional leadership scores. The results indicate that higher scores on one scale tend to correspond to higher scores on the other (see Figure 1). Conversely, laissez-faire scores showed a moderate negative correlation (r = −0.339, *p* < 0.01) with transformational leadership scores, indicating that higher laissez-faire scores were associated with lower levels of transformational leadership. Lastly, a weak positive correlation (r = 0.201, *p* < 0.05) was identified between transactional and laissez-faire leadership scores.

## 4. Discussion

This study examined the leadership styles of nurses from a behavioural configuration perspective, finding that a transformational style was predominant, accompanied by transactional practices and occasional laissez-faire leadership. This pattern is consistent with recent research identifying transformational leadership as the most prevalent style in contemporary nursing contexts. In complex healthcare settings, transformational leadership has been consistently associated with positive environments for teams and patient outcomes [1,18]. It is argued by some authors that the implementation of transformational leadership styles in nurse management can serve as a promising strategy for nursing leaders to enhance the alignment between individuals and organisations, thereby stabilising nursing teams [2].

Transformational leadership is a common style of leadership, and research has identified links to key skills for dealing with new challenges, such as delivering quality, innovation and teamwork in healthcare [1,18].

Transformational dimensions such as individual consideration and inspirational motivation stood out in the sample, which aligns with evidence that these behaviours promote greater professional engagement, occupational well-being, and creativity in clinical practice [6,24].

The coexistence of transactional leadership, with an emphasis on contingent reinforcement, reflects the complementary nature of leadership styles. Recent studies reaffirmed that effective healthcare leaders tend to integrate transactional behaviours—especially setting clear expectations and recognising performance—within a broader transformational framework [7,25]. This pattern suggests that leadership behaviours may be combined in practice, with transactional elements functioning alongside transformational behaviours rather than in opposition to them.

In contrast, laissez-faire leadership was low, as has been previously observed in other studies of nursing leadership [3,8]. Contemporary literature highlights that passive or non-existent leadership styles are often associated with more negative perceptions of the working environment and lower team performance [1,5]. The lower prevalence of this style in the sample is consistent with the generally active leadership profile observed in this leadership-engaged group.

The gender-based analysis revealed no statistically significant differences in the leadership dimensions assessed. This finding aligns with recent research that challenges the notion of gender as a standalone factor in shaping leadership styles, emphasising that variables such as training, organisational context and professional competence hold more significant influence [26]. In addition, age demonstrated weak and sporadic associations with certain dimensions, thereby reinforcing the notion that isolated demographic characteristics account for only a minor proportion of the variability in leadership styles [27]. These results should be interpreted cautiously given their limited magnitude and the lower reliability of some subscale measures in the present sample.

Some differences were observed between nurse managers and specialist nurses, with managers showing higher mean values in selected transformational indicators. Although these findings may suggest differences linked to role-related experience and responsibilities, they should be interpreted cautiously, particularly at the subscale level, given the exploratory analyses and the lower reliability of some subscale measures. This pattern is broadly consistent with the view that formal management experience may support the development of more structured and strategic leadership behaviours [28]. Recent studies demonstrate that leadership training opportunities and practical management experiences can enhance leaders’ ability to operate in complex environments and promote sustained change [28].

In line with the study objective of identifying leadership styles among nurses within a management-oriented professional context, the findings demonstrate that leadership styles coexist in complementary dimensional patterns, consistent with the conceptual assumptions of the Full Range Leadership Model. The predominance of transformational leadership, combined with complementary transactional elements and minimal passive–avoidant expression, supports a dimensional interpretation of leadership consistent with contemporary behavioural frameworks. Overall, these findings provide contextual descriptive evidence on leadership patterns within a Portuguese nursing sample engaged in management and leadership roles, and may help inform future leadership development initiatives in comparable contexts.

In addition, although the 36 MLQ-5X items analysed in this study showed acceptable overall internal consistency, some individual subscales demonstrated low to modest reliability, suggesting that subscale-level findings should be interpreted with caution.

These findings should be interpreted in light of the study design and sampling strategy. Because participants were recruited through a leadership-focused professional association and data were obtained through self-report, the results likely reflect a selected subgroup of nurses with greater engagement in leadership and may also be affected by social desirability and non-response bias.

This study adds descriptive evidence to the literature by showing that leadership dimensions may coexist within nursing management contexts in a way that is consistent with the Full Range Leadership Model.

### 4.1. Strengths and Limitations of the Work

This study has a relatively large and diverse sample, covering different management levels and professional categories. To ensure reliable and valid measurements, it is essential to use an internationally validated instrument, such as the MLQ (Multifactor Leadership Questionnaire). A detailed quantitative analysis was conducted, incorporating significance tests, effect sizes and correlations, enabling a robust assessment of the magnitude of differences.

This study has several limitations that should be acknowledged. The main limitation is the selective nature of the sample. Participants were recruited through a professional nursing association specifically focused on management and leadership, which means the sample likely reflects a subgroup of nurses with greater interest, training, and engagement in leadership than the broader nursing population. This introduces a substantial risk of selection bias and is the main reason why the findings should not be generalised to nurses more broadly. In addition, the use of a non-probability convenience sample further limits representativeness. The response rate was modest, and non-response bias cannot be excluded. The use of self-reported data may also have introduced social desirability bias and common method bias. Although the MLQ-5X is a well-established instrument [11,12], validity was not formally reassessed in the present sample. While the 36 items analysed in this study showed acceptable overall internal consistency, and some broader dimensions demonstrated stronger reliability, several individual subscales showed low to modest internal consistency, which may limit the precision of subscale-level interpretation. The analysis did not examine the impact of ethnicity on leadership approaches. Finally, the cross-sectional design precludes causal inference and does not allow conclusions about temporal changes in leadership patterns.

### 4.2. Recommendations for Further Research

This study provides a solid foundation for the recommendation of longitudinal studies. These studies will assess the evolution of leadership styles throughout careers and their impact on the quality of care. Another recommended study is a combined assessment of self-reporting and team perception to reduce social desirability bias. Research could also be conducted to explore interactions between leadership and other contextual factors, such as organisational culture, human resources and health policies. It will be important to develop training interventions focused on transformational leadership and evaluate their effects on the health outcomes and well-being of teams.

## 5. Conclusions

The present study aimed to examine the distribution and interrelationships of leadership dimensions among nurses within the framework of the Full Range Leadership Model. The findings indicate that transformational leadership represented the predominant pattern within the sample, complemented by moderate levels of transactional behaviours and minimal expression of passive–avoidant leadership.

These results suggest that leadership among nurses in management-oriented contexts tends to be characterised by an active and relational profile, emphasising team motivation, professional development, and supportive working environments. The coexistence of transformational and transactional behaviours indicates that leadership practices are not expressed as mutually exclusive styles but rather as complementary and interrelated dimensions, consistent with the conceptual assumptions of the Full Range Leadership Model.

By situating leadership behaviours within this theoretical framework, the study offers descriptive insight into how leadership dimensions may coexist and interact in this specific nursing context. Although the cross-sectional nature of the study does not allow causal inference, and the sampling strategy requires caution in generalising the findings, the patterns identified provide useful descriptive insights into leadership behaviours within nursing management contexts. Understanding these leadership configurations may inform the development of leadership education and professional development initiatives, particularly in relation to transformational and transactional leadership dimensions in healthcare settings.

## Figures and Tables

**Table 1:** Descriptive Characteristics of the Study Sample.

Variable	Sample (*n* = 141)
Gender (%)	
Female	110 (78.0%)
Male	31 (22.0%)
Age (years)	
Mean (SD)	52.50 (7.92)
Median	53.00
Min-Max	30–66
Academic qualifications (%)	
Bachelor’s degree	53 (37.6%)
Master’s degree	78 (55.3%)
Doctorate	10 (7.1%)
Professional competencies (%) *	
Postgraduate Degree in Management/Administration/Leadership	127 (90.1%)
Title of Specialist by the Order of Nurses	136 (96.5%)
Increased competence in management by the Order of Nurses	89 (63.1%)
Speciality, where applicable (%)	
Surgical Medical Nursing	40 (28.4%)
Rehabilitation Nursing	34 (24.1%)
Public, Community and Family Nursing	24 (17.0%)
Child and Paediatric Health Nursing	18 (12.8%)
Mental Health and Psychiatric Nursing	10 (7.1%)
Maternal and Obstetric Health Nursing	9 (6.4%)
Work experience (years)	
Mean (SD)	29.96 (7.79)
Median	30
Min-Max	10–44
Professional experience in management/leadership (years)	
Mean (SD)	12.74 (8.85)
Median	11
Min-Max	0–32
Region of the country	
Azores Island	2 (1.4%)
Alentejo	12 (8.5%)
Algarve	2 (1.4%)
Centre Region	18 (12.8%)
Lisbon and Tagus Valley	39 (27.7%)
Madeira Island	2 (1.4%)
North	66 (46.8%)
Professional practice sector	
Public	134 (95.0%)
Private	4 (2.8%)
Public–Private	3 (2.1%)
Professional practice context	
Hospital healthcare	97 (68.8%)
Primary healthcare	29 (20.6%)
Academy	6 (4.3%)
Others	9 (6.4%)

* Nurses may have completed more than one professional training programme.

**Table 2:** Descriptive Statistics of Leadership Dimensions and Subdimensions.

	Mean	SD	Min	Max	*n* (%)
Transformational Leadership	3.17	0.38	2.25	3.85	132 (93.6%)
Attributes of Idealised Influence	2.83	0.50	1.50	4.00	
Behaviours of Idealised Influence	3.25	0.47	2.00	4.00	
Inspirational Motivation	3.23	0.56	1.50	4.00	
Intellectual Stimulation	3.14	0.46	2.00	4.00	
Individual Consideration	3.40	0.48	2.00	4.00	
Transactional Leadership	2.51	0.46	1.25	3.75	9 (6.4%)
Contingent Reward	2.86	0.57	1.25	4.00	
Management-by-Exception (Active)	2.16	0.64	0.50	3.75	
Passive–Avoidant Leadership	0.83	0.50	0.00	3.25	0 (0%)
Management-by-Exception (Passive)	1.01	0.53	0.00	3.00	
Laissez-faire	0.65	0.55	0.00	3.50	

**Table 3:** Mean differences in leadership style scores by gender.

	Gender	Mean	SD	Difference	t	*p*-Value
Transformational Leadership						
	Female	3.165	0.371	−0.017	−0.224	0.833
	Male	3.182	0.408			
Attributes of Idealised Influence	Female	2.825	0.504	−0.014	−0.134	0.894
	Male	2.839	0.502			
Behaviours of Idealised Influence	Female	3.227	0.467	−0.103	−1.088	0.282
	Male	3.331	0.467			
Inspirational Motivation	Female	3.230	0.537	0.012	0.092	0.927
	Male	3.218	0.657			
Intellectual Stimulation	Female	3.143	0.460	0.030	0.316	0.753
	Male	3.113	0.474			
Individual Consideration	Female	3.400	0.497	−0.011	−0.125	0.901
	Male	3.411	0.426			
Transactional Leadership						
	Female	2.494	0.457	−0.054	−0.575	0.568
	Male	2.548	0.464			
Contingent Reward	Female	2.846	0.567	−0.050	−0.415	0.680
	Male	2.895	0.594			
Management-by-Exception (Active)	Female	2.143	0.638	−0.058	−0.437	0.664
	Male	2.202	0.663			
Passive–Avoidant Leadership						
	Female	0.853	0.528	0.116	1.420	0.160
	Male	0.738	0.356			
Management-by-Exception (Passive)	Female	1.039	0.544	0.135	1.379	0.173
	Male	0.903	0.464			
Laissez-faire	Female	0.668	0.588	0.096	1.097	0.276
	Male	0.573	0.372			

**Table 4:** Pearson correlations between leadership style and age.

	r (Pearson)	*p*-Value
Transformational Leadership	−0.004	0.959
Attributes of Idealised Influence	−0.174 *	0.039
Behaviours of Idealised Influence	0.107	0.208
Inspirational Motivation	0.036	0.670
Intellectual Stimulation	0.050	0.558
Individual Consideration	−0.029	0.729
Transactional Leadership	0.015	0.860
Contingent Reward	0.064	0.448
Management-by-Exception (Active)	−0.036	0.671
Passive–Avoidant Leadership	−0.163	0.053
Management-by-Exception (Passive)	−0.231 **	0.006
Laissez-faire	−0.073	0.393

* Correlation is significant at the 0.05 level (2-tailed); ** Correlation is significant at the 0.01 level (2-tailed).

**Table 5:** Mean differences in leadership style scores by professional category.

	Professional Category	Mean	SD	Difference	t	*p*-Value	d (Cohen) *
Transformational Leadership							
	Manager Nurse	3.23	0.33	0.160	2.15	0.018	0.43
	Specialist Nurse	3.07	0.43				
Attributes of Idealised Influence	Manager Nurse	2.86	0.48	0.073	0.77	0.446	N/A
	Specialist Nurse	2.79	0.53				
Behaviours of Idealised Influence	Manager Nurse	3.29	0.45	0.147	1.72	0.090	N/A
	Specialist Nurse	3.15	0.46				
Inspirational Motivation	Manager Nurse	3.30	0.47	0.230	1.98	0.053	N/A
	Specialist Nurse	3.07	0.69				
Intellectual Stimulation	Manager Nurse	3.21	0.45	0.220	2.60	0.011	0.49
	Specialist Nurse	2.99	0.46				
Individual Consideration	Manager Nurse	3.45	0.43	0.129	1.29	0.200	N/A
	Specialist Nurse	3.32	0.58				
Transactional Leadership							
	Manager Nurse	2.51	0.44	−0.021	−0.23	0.816	N/A
	Specialist Nurse	2.53	0.50				
Contingent reward	Manager Nurse	2.90	0.57	0.103	0.94	0.351	N/A
	Specialist Nurse	2.80	0.61				
Management-by-Exception (Active)	Manager Nurse	2.12	0.65	−0.145	−1.22	0.224	N/A
	Specialist Nurse	2.27	0.63				
Passive–Avoidant Leadership							
	Manager Nurse	0.74	0.42	−0.273	−2.66	0.010	−0.55
	Specialist Nurse	1.01	0.61				
Management-by-Exception (Passive)	Manager Nurse	0.91	0.46	−0.308	−2.89	0.005	−0.59
	Specialist Nurse	1.22	0.62				
Laissez-faire	Manager Nurse	0.57	0.47	−0.238	−2.11	0.039	−0.55
	Specialist Nurse	0.81	0.67				

N/A = not applicable. * Cohen’s d only shown for statistically significant differences.

**Table 6:** Mean differences in leadership style scores by academic qualification.

	Academic Qualification	Mean	SD	Difference	t	*p*-Value	d (Cohen) *
Transformational Leadership							
	PhD	3.37	0.41	0.22	1.72	0.089	N/A
	Other	3.15	0.37				
Attributes of Idealised Influence	PhD	2.90	0.53	0.08	0.47	0.640	N/A
	Other	2.82	0.50				
Behaviours of Idealised Influence	PhD	3.60	0.49	0.38	2.50	0.014	0.82
	Other	3.22	0.46				
Inspirational Motivation	PhD	3.55	0.57	0.35	1.90	0.059	N/A
	Other	3.20	0.56				
Intellectual Stimulation	PhD	3.33	0.43	0.21	1.34	0.181	N/A
	Other	3.12	0.46				
Individual Consideration	PhD	3.45	0.40	0.05	0.32	0.747	N/A
	Other	3.40	0.49				
Transactional Leadership							
	PhD	2.48	0.42	−0.03	−0.22	0.824	N/A
	Other	2.51	0.46				
Contingent Reward	PhD	2.95	0.55	0.10	0.54	0.592	N/A
	Other	2.85	0.57				
Management-by-Exception (Active)	PhD	2.00	0.51	−0.17	−0.80	0.427	N/A
	Other	2.17	0.65				
Laissez-Faire Leadership							
	PhD	0.66	0.43	−0.18	−1.09	0.276	N/A
	Other	0.84	0.50				
Management-by-Exception (Passive)	PhD	0.73	0.34	−0.30	−1.77	0.078	N/A
	Other	1.03	0.54				
Laissez-Faire	PhD	0.60	0.57	−0.05	−0.28	0.790	N/A
	Other	0.65	0.55				

N/A = not applicable. * Cohen’s d only shown for statistically significant differences.

## Data Availability

The datasets generated and analysed during the current study are available from the corresponding author upon reasonable request. The data are not publicly available due to ethical and confidentiality restrictions; however, access to anonymized data may be granted upon request.

## Public Involvement Statement

Patients, carers, and members of the public were not involved in the design, conduct, reporting, or dissemination of this research.

## Guidelines and Standards Statement

For the purposes of this study, the STROBE reporting guideline [19] was used to draft this manuscript, and the STROBE reporting checklist [20] was used when editing.

## Abbreviations

The following abbreviations are used in this manuscript:

MLQ	Multifactor Leadership Questionnaire
SPSS	Statistical Package for the Social Sciences
FRLM	Full Range Leadership Model
STROBE	Strengthening the Reporting of Observational Studies in Epidemiology
